# Emotion Detection from EEG Signals Using Machine Deep Learning Models

**DOI:** 10.3390/bioengineering11080782

**Published:** 2024-08-02

**Authors:** João Vitor Marques Rabelo Fernandes, Auzuir Ripardo de Alexandria, João Alexandre Lobo Marques, Débora Ferreira de Assis, Pedro Crosara Motta, Bruno Riccelli dos Santos Silva

**Affiliations:** 1 Programa de Pós-Graduação em Engenharia de Telecomunicações, Instituto Federal do Ceará (IFCE), Fortaleza 60040-215, Brazil; auzuir@ifce.edu.br; 2 Laboratory of Applied Neurosciences (LAN), University of Saint Joseph, Saint Joseph 999078, Macau; alexandre.lobo@usj.edu.mo; 3 Programa de Pós-Graduação em Engenharia de Teleinformática, Universidade Federal do Ceará, Fortaleza 60455-760, Brazil; debora.ferreira@lesc.ufc.br (D.F.d.A.); bruno@lesc.ufc.br (B.R.d.S.S.); 4 Programa de Pós-Graduação em Engenharia Biomédica, Universidade Federal do Rio de Janeiro, Rio de Janeiro 21941-598, Brazil; pedro.motta@lesc.ufc.br

**Keywords:** deep learning, emotion detection, emotion recognition, electroencephalogram, graph convolutional neural networks, machine learning

## Abstract

Detecting emotions is a growing field aiming to comprehend and interpret human emotions from various data sources, including text, voice, and physiological signals. Electroencephalogram (EEG) is a unique and promising approach among these sources. EEG is a non-invasive monitoring technique that records the brain’s electrical activity through electrodes placed on the scalp’s surface. It is used in clinical and research contexts to explore how the human brain responds to emotions and cognitive stimuli. Recently, its use has gained interest in real-time emotion detection, offering a direct approach independent of facial expressions or voice. This is particularly useful in resource-limited scenarios, such as brain–computer interfaces supporting mental health. The objective of this work is to evaluate the classification of emotions (positive, negative, and neutral) in EEG signals using machine learning and deep learning, focusing on Graph Convolutional Neural Networks (GCNN), based on the analysis of critical attributes of the EEG signal (Differential Entropy (DE), Power Spectral Density (PSD), Differential Asymmetry (DASM), Rational Asymmetry (RASM), Asymmetry (ASM), Differential Causality (DCAU)). The electroencephalography dataset used in the research was the public SEED dataset (SJTU Emotion EEG Dataset), obtained through auditory and visual stimuli in segments from Chinese emotional movies. The experiment employed to evaluate the model results was “subject-dependent”. In this method, the Deep Neural Network (DNN) achieved an accuracy of 86.08%, surpassing SVM, albeit with significant processing time due to the optimization characteristics inherent to the algorithm. The GCNN algorithm achieved an average accuracy of 89.97% in the subject-dependent experiment. This work contributes to emotion detection in EEG, emphasizing the effectiveness of different models and underscoring the importance of selecting appropriate features and the ethical use of these technologies in practical applications. The GCNN emerges as the most promising methodology for future research.

## 1. Introduction

Human emotion detection and recognition are crucial in advancing human interactions and technological systems. In recent years, the application of signal processing techniques, machine learning, and artificial intelligence has shown promise in analyzing electroencephalography (EEG) signals for emotion classification [1]. EEG is an electrophysiological recording of brain activity that provides information about an individual’s emotional states, offering a window into understanding the neural responses underlying different emotional stimuli [2].

Emotion recognition focuses on various modalities, such as audiovisual expressions, body language, and physiological signals. Compared to other modalities, physiological signals, such as electroencephalography (EEG), electrocardiography (ECG), and electromyography (EMG), have the advantage of being difficult to hide or disguise. In recent years, due to the rapid development of non-invasive, easy-to-use, and more affordable EEG recording devices compared to other acquisition techniques, such as Magnetic Resonance Imaging (MRI) and Magnetoencephalography (MEG), EEG-based emotion recognition has received increasing research attention [3] and applications (Zhong, Wang, and Miao) [4]. While EEG has limited spatial resolution and requires many electrodes placed in various locations on the head, it provides adequate time resolution, allowing researchers to study phase changes in response to emotional stimuli. Additionally, EEG is non-invasive, fast, and inexpensive, making it a more common method for studying the brain’s responses to emotional stimuli [5].

Various efforts have been made to explore the use of machine learning models, such as Logistic Regression (LR), k-nearest Neighbors (KNN), Support Vector Machines (SVM), Deep Neural Networks (DNN), and Dynamic Graph Convolutional Neural Networks (DGCNN), in analyzing emotions in EEG signals; however, many challenges persist [6,7]. The overlap of brain activation patterns between different emotions, inter-individual variability, and inherent noise in EEG signals are just a few examples of the obstacles that must be overcome to achieve accurate and robust classification [8].

The theoretical background of this research is based on the application of machine learning and deep learning techniques, which have emerged as a promising approach for advancing emotion recognition from EEG signals [9]. LR models the probability of an emotional state as a function of the EEG features, while KNN classifies emotions based on the similarity of EEG patterns to those of labeled examples. SVM, on the other hand, can capture nonlinear relationships between EEG features and emotional states by using kernel functions to map the data into a higher-dimensional space. To address these limitations, deep learning approaches have gained significant attention in the field of EEG-based emotion recognition [10]. Deep Neural Networks (DNNs) are a class of deep learning models that can automatically learn hierarchical representations from raw EEG signals, enabling the capture of complex nonlinear patterns associated with emotional processing in the brain. By stacking multiple layers of artificial neurons, DNNs can extract increasingly abstract and meaningful features from the EEG data, leading to superior performance in emotion classification tasks compared to traditional machine learning models [11].

Furthermore, the incorporation of graph-based deep learning techniques, such as Graph Convolutional Neural Networks (GCNNs), has demonstrated the ability to model the spatial relationships between EEG electrodes. EEG signals can be represented as a graph, where the electrodes are the nodes and the connections between them reflect the spatial dependencies in the brain [4]. GCNNs leverage this graph structure to learn features that capture the complex spatial and temporal dynamics of EEG signals, providing a more comprehensive understanding of the neural correlates of emotions.

Emotion classification in EEG signals represents a multidisciplinary challenge that involves the intersection of digital signal processing, machine learning, and neuroscience. The importance of emotion classification using EEG signals is significant for multiple applications. However, there is a lack of scientific publications comprehensively testing and comparing various models to solve this problem. To address this gap, the main objective of this research is to evaluate the effectiveness of different machine learning models, ranging from more conventional techniques like Logistic Regression, K-Nearest Neighbors, and Support Vector Machines to advanced deep learning approaches such as Deep Neural Networks and Graph Convolutional Neural Networks (GCNNs). By applying these diverse models to analyze the key EEG signal attributes, the study aims to deepen the understanding of the complex relationships between brain activity patterns and emotional states. This work seeks to identify the best-performing models and contribute to the evolution of the field, with the potential to positively impact society and technology.

The novelty of this research lies in its comprehensive and comparative analysis of a wide range of machine learning and deep learning approaches, including the innovative use of GCNNs, to advance emotion detection from EEG signals and address the gaps in the existing literature.The use of DGCNNs in this context is an innovative approach, leveraging the network’s ability to learn hierarchical and dynamic representations and capture the temporal evolution of neural patterns.

## 2. Related Works

The electroencephalogram (EEG) finds wide application across various domains. Its role stands out notably in the realm of emotion recognition. By identifying brain patterns, EEG translates emotional nuances such as happiness, sadness, and stress, as identified by Liu et al. [6] and Zheng, Zhu, and Lu [12]. Thus, EEG bridges the gap between brain electrical activity and the complexity of human emotions, driving innovations across diverse fields.

Zheng, Zhu, and Lu (2019) [12] address the temporal stability of EEG patterns over time. The study evaluates feature extraction, selection, smoothing, and pattern classification methods. The authors utilized the DEAP and SEED datasets. The Discriminative Graph Regularized Extreme Learning Machine (GELM) algorithm with features of differential entropy achieves the best average accuracy compared to the results of KNN, Logistic Regression, and SVM classifiers. The findings demonstrate that stable patterns exhibit consistency across sessions, revealing specific characteristics for positive, neutral, and negative emotions in different brain regions and frequency bands.

Gan et al. (2019) [9] explores deep learning techniques to identify emotional states from voice signals and facial expressions using frequency band peculiarities. Additionally, the study analyzes the application of differential entropy (DE) to enhance accuracy in signal feature extraction. The study highlights the capability of deep neural networks to analyze multiple modalities to improve cross-cultural emotion identification.

Recently, Li et al. (2021) [13] proposed using RNNs to learn spatial topological relationships among channels by scanning electrodes vertically and horizontally. However, the model must effectively capture the complex spatial relationships present in EEG signals.

Yongqiang [14] (2021) proposed the ECLGCNN model, a fusion of LSTM and GCNN, to enhance emotion recognition. This method extracts differential entropy (DE) from segments to form a feature cube. Multiple GCNNs extract information from the graphical domain of each cube, while LSTM cells memorize changes in the relationships between EEG channel pairs and extract temporal information.

Zhong, Wang, and Miao (2022) [4] proposes a regularized graph neural network for emotion recognition. The model captures the relationships between local and global channels in EEG signals. The adoption of graphs demonstrates a step forward by effectively incorporating the complex spatial relationships present in EEG data.

The recent work of Zhang et al. (2022) [15] introduces the concept of the Graph Convolutional Broad Network (GCB-net), which employs regular convolution to explore deep information, abstracting high-level features from graph representations. Additionally, it incorporates a Broad Learning System, acting as an adaptive layer to select appropriate features. This approach aims to capture high- and low-frequency information from EEG signals, enabling advancements in classification through a broad network.

Zhang et al. (2023) [10] enhanced the DGCNN model by introducing a dispersion constraint in the graph representation G and proposing a solution for minimization with this constraint (SparseDGCNN). This method demonstrated superior performance to its non-sparse version, highlighting the effectiveness of introducing dispersion in the irregular graph connectivity patterns of EEG signals for emotion classification. The SparseDGCNN approach combines the DGCNN architecture with sparsity techniques, allowing for the representation of temporal and spatial patterns in EEG signals while reducing computational complexity.

Jeong, Kim, and Kim (2023) [16] proposed a hierarchical neural network with self-attention, extracting local and global features for EEG-based emotion recognition using a Hierarchical Space-temporal Context Feature Learning Model (HSCFLM). They evaluated the DEAP, MAHNOB-HCI, and SEED datasets. Pre-processing involved band-pass filtering, resolution reduction, blink artifact removal, segmentation, and spectral analysis for each frontal, temporal, central, parietal, and occipital region. Methods such as CNN, LSTM, BGRU, CRNN, and HA-BGRU were compared, where multichannel EEG signals were applied to each neural network without dividing the brain into regions. Proposed methods like R2G HA-BGRU, R2GTF, R2G HAS-BGRU, and HSCFLM divided the brain into nine defined regions, grouping corresponding electrodes by region. The best results were obtained with the MAHNOB-HCI dataset, achieving two-level classification with an accuracy for valence, arousal, and dominance of 93.3%, 91.6%, and 92.8%, respectively. The three-level classification with the same dataset reached 88.9%, 89.1%, and 89.4%.

Zhang, X., et al. (2023) [17] developed a novel self-training maximum classifier discrepancy (SMCD) method for EEG signal emotion recognition. The proposed approach leverages a feature generator and two classifiers to detect the decision boundary of a particular task, thereby maximizing the discrepancies between the two classifiers’ outputs. This method effectively deals with domain transfer problems by using unlabeled test data to fully utilize knowledge from the new subject and reduce the domain gap. Additionally, a 3D Cube is constructed to incorporate the spatial and frequency information of the EEG data, creating input features for a Convolutional Neural Network (CNN). Extensive experiments on the SEED and SEED-IV datasets demonstrate the superior performance of the proposed method over state-of-the-art methods.

The authors Dwivedi, Verma, and Taran (2024) [18] transformed EEG signals from the GAMEEMO dataset into images using time–frequency representation techniques, achieving the best results with Smoothed Pseudo Wigner–Ville Distribution (SPWVD). Subsequently, they applied the GoogleNet network to extract features and optimized its parameters. Moreover, they employed Bayesian optimization (BO) to automate the selection of CNN hyperparameters to predict four emotional states. The method achieved an accuracy of 84.2%, and the AUC for the boring, horror, calm, and funny classes were 0.9712, 0.9596, 0.9597, and 0.9625, respectively.

Fan et al. (2024) [19] proposed a dual-module approach using an enhanced capsule network and long short-term residual memory (ICaps-ResLSTM). This approach consists of ICapsNet to capture spatial information from EEG signals and ResLSTM to extract high-level temporal information, obtaining high-resolution spatiotemporal features. The average accuracy of 10-fold for arousal, valence, and dominance in the DREAMER dataset reached 94.97%, 94.71%, and 94.96%, respectively.

Roshdy et al. (2024) [20] evaluated the combination of facial expression analysis from DeepFace with CNN based on EEG signals. In this approach, raw EEG data undergo processing to generate a heat map of brain activity. The results for happy and sad emotions achieved an efficiency of 91.21% when integrating CNN with DeepFace CNN.

These approaches can potentially deepen our understanding of the complex interactions between EEG channels and provide significant gains in emotion classification accuracy Rajwal and Aggarwal (2023) [21]. The convergence of various studies emphasizes the importance of exploring graph neural network architecture to enhance EEG signal analysis, offering an exciting perspective for future advances at the interface of neuroscience, signal processing, and machine learning.

In an increasingly user-centric context, interpreting and reacting to human emotions becomes crucial for developing more effective interfaces and interactions. Understanding emotional responses allows the creation of more empathetic technologies which are capable of dynamically adapting to users’ needs and emotional states. Therefore, this work evaluates the main attributes of the EEG signal and analyzes the classification of emotions using machine learning and deep learning. Table 1 compiles and presents the results of each of the referenced studies.

## 3. Materials and Methods

This study aims to investigate EEG data using machine learning techniques for pattern recognition and extracting emotion-relevant information. In the following sections, the methodological aspects applied, the dataset used, research factors, and measurement variables will be described.

### 3.1. EEG Datasets

The choice of EEG dataset plays a crucial role in conducting research and studies related to emotion recognition. However, obtaining suitable EEG datasets can be challenging due to several inherent difficulties.

Firstly, the availability of emotion-specific EEG datasets is relatively limited compared to other research areas [2]. This is due to EEG data’s sensitive and restricted nature, which are collected directly from individuals’ brains. As a result, researchers may face difficulties finding appropriate datasets that align with their specific research objectives.

Furthermore, these datasets often have size, security, and cost limitations. EEG data are voluminous regarding the number of samples and dimensions, which can restrict access to larger datasets [22]. Additionally, the security and privacy of participants involved in EEG data collection are paramount concerns, often requiring stringent protocols for data acquisition, storage, and sharing [1]. This can further complicate the accessibility of publicly available EEG datasets. Moreover, preparing and curating EEG data can be costly in terms of human and financial resources, as EEG data collection, processing, and labeling require technical expertise and specialized equipment.

The choice of an EEG dataset is crucial for the success of emotion recognition research. Despite the abovementioned challenges in obtaining such data, several public and private datasets have been instrumental in advancing the field [23]. These datasets provide researchers with a rich source of brain signals recorded during different types of emotional stimuli and activities, enabling the analysis and development of robust and effective emotion recognition algorithms.

Among the notable EEG datasets, it is possible to highlight the following:

- *SEED* (*SJTU Emotion EEG Dataset*)—[12]
- *DEAP* (*Database for Emotion Analysis using Physiological Signals*) *Dataset*—[24]
- *DREAMER* (*Database for Emotion Analysis using Physiological Signals) Dataset*—[25]
- *TUH EEG* (*Temple University Hospital EEG) Dataset*—[26]
- *MPI LEMON* (*Max Planck Institute for Biological Cybernetics Linear EEG data*) Dataset—[27]
- *MAHNOB HCI* (*Multimedia Analysis and Human-Computer Interaction*)—[28]

These datasets vary in size, format, quality, and purpose, but they all provide valuable information for analyzing EEG patterns associated with different emotions.

For example, the SEED (SJTU Emotion EEG Dataset) contains EEG data from multiple subjects experiencing different emotional stimuli, making it a valuable resource for training and testing emotion recognition algorithms [12]. The DEAP and the DREAMER, on the other hand, present multimodal physiological signals, including EEG, collected during the presentation of audiovisual stimuli designed to evoke different emotions [24,25]. The TUH EEG Dataset and the MPI LEMON Dataset provide clinical EEG data collected from patients in hospital settings, enabling the exploration of emotions in medical contexts [26]. The MAHNOB HCI Dataset was designed to capture a wide range of human emotions, collecting data from subjects exposed to varied emotional stimuli such as images, videos, and music. It contains detailed records of EEG signals from each subject, enabling the analysis of variations in brain patterns during different emotional states [28].

Among the various datasets available for research in emotion recognition, the SEED (SJTU Emotion EEG Dataset) stands out as a notable option due to its distinct characteristics and peculiar advantages. SEED provides a controlled environment for acquiring EEG data related to emotions, encompassing a diverse and well-defined range of emotional stimuli, resulting in a comprehensive spectrum of captured emotional responses. This, in turn, enriches the intrinsic variety and representativeness of the dataset. Additionally, the dataset features high-quality EEG signals, with rigorous control over interference and noise, ensuring the results’ reliability. The precise spatial resolution of the electrodes used in SEED is worth noting, enabling the accurate identification of brain activities in specific regions. This feature is crucial in understanding the correlations between brain patterns and emotional states, fostering more thorough analyses and deeper correlations. Therefore, the selection of SEED as the basis for this dissertation is grounded in these advantages, which allow for the in-depth exploration of the complex interactions between the brain and emotions through machine learning techniques.

### 3.2. SEED

The SJTU Emotion EEG Dataset (SEED) is a dataset that captures brain responses associated with different emotional states. EEG data were collected from fifteen subjects (seven males and eight females, mean age = 23.27). These EEG data were obtained through auditory and visual stimuli in the form of excerpts from Chinese emotional movies. While participants watched 15 four-minute movie excerpts with three types of emotion (positive, negative, and neutral), their EEG data were recorded by an ESI NeuroScan system with 62 channels at a sampling rate of 1000 Hz. The EEG data were downsampled to 200 Hz and manually checked to remove EOG and EMG artifacts [29]. EEG recordings were captured for each subject over three chronologically disconnected sessions, and each session repeated the same experiment. Additionally, SEED does not contain arousal information; therefore, it only recognized positive, neutral, and negative emotions in SEED. After preprocessing with a band-pass filter (between 0.3 and 50 Hz), five features (DE, PSD, DASM, RASM, and DCAU) were extracted with a 1-second window in five bands: delta (0.5–4 Hz), theta (4–7 Hz), alpha (8–13 Hz), beta (14–30 Hz), and gamma (>30 Hz) bands.

### 3.3. EEG Pre-Processing

It is imperative to understand the intricate feature extraction process in order to understand the complexities of analyzing EEG signals in emotion recognition. This process is pivotal in unraveling significant insights embedded within EEG data, facilitating subsequent classification and recognition tasks. The multifaceted nature of EEG signals necessitates a meticulous approach across various domains, including time and frequency, each offering unique advantages and challenges. In the following discussion, the fundamental principles and methodologies in extracting features from EEG signals are explored, shedding light on the diverse strategies employed to decipher the intricate interplay between brain dynamics and emotional states.

#### 3.3.1. Feature Extraction

The analysis of EEG signals is a complex task due to the multidimensional and dynamic nature of these signals [30]. Therefore, proper feature extraction is crucial to highlight important information that can be used in subsequent processes of classification and emotion recognition [31].

Feature extraction is conducted in three main dimensions: the time domain, the frequency domain, and the combination of both domains. In the time domain, various statistical attributes are directly extracted from EEG signals. Among these attributes, the mean, standard deviation, kurtosis, and skewness stand out, providing information about central tendency, dispersion, and signal shape [32]. Additionally, temporal features such as zero-crossing rate and signal energy are also used to understand aspects of temporal variation.

In the frequency domain, techniques such as the Fourier Transform are applied to map EEG signals from the time domain to the frequency domain. This allows the analysis of spectral components present in the signals, revealing patterns of activity in different frequency bands [22]. Spectral features such as power spectral density (PSD) and the ratio between frequency bands are extracted to capture the distribution of energy across the frequency spectrum [7].

Combining features extracted from both the time and frequency domains provides a comprehensive representation of EEG signals, allowing for the identification of discriminative patterns related to emotions [14]. This feature extraction step is crucial for feeding the classification algorithms employed, enabling the development of robust emotional recognition models from EEG signals.

In the time domain, advantages include directly representing EEG signals in their raw form, allowing for the direct visualization of temporal variations [32]. Features such as peak amplitudes and durations can be observed, preserving important information about temporal dynamics. However, there are disadvantages, such as susceptibility to noise, as EEG signals can be affected by various types of interference. Additionally, analyzing complex patterns in temporal signals can be challenging, significantly when multiple brain activities are overlapping [5].

In the frequency domain, the main advantage is spectral characterization, which enables the identification of activity patterns in different frequency bands such as delta, theta, alpha, beta, and gamma, providing information about specific brain activities [30]. The detection of spectral components such as frequency peaks is also possible. However, there is a disadvantage associated with the loss of temporal information, as the Fourier transformation does not preserve the temporal sequence of events. Additionally, the overlap of different brain activities in the same frequency band can make it difficult to distinguish between these activities [32].

The time–frequency domain combines the advantages of the previous domains, allowing for the visualization of how spectral features evolve [7]. It is suitable for detecting rapid changes in spectral features, such as transient frequency peaks, indicating dynamic changes in the signal. However, it presents limitations, such as limited resolution due to the Heisenberg uncertainty principle, which imposes a trade-off between temporal and frequency resolution. Additionally, transforming the signal into the time–frequency domain requires higher computational complexity [30].

In summary, the choice of EEG analysis domain depends on the objectives and context of the analysis. The time domain is suitable for studying rapid and complex events, and the frequency domain is useful for characterizing different brain activities. In contrast, the time–frequency domain combines the former’s advantages but requires the consideration of resolution and computational complexity [8].

The SJTU Emotion EEG Dataset (SEED) provides some intrinsic characteristics of electroencephalography (EEG) signals that can be explored as measurement variables for the analysis of relevant patterns in identifying participants’ emotions. These characteristics include the following:

- Differential entropy (DE);
- Power spectral density (PSD);
- Differential asymmetry (DASM);
- Rational asymmetry (RASM);
- Asymmetry (ASM);
- Differential causality (DCAU).

The rationale for including these specific EEG features is that the dataset already includes them, but also because each measurement variable has a unique characteristic related to the EEG signal. The power spectral density (PSD) describes power distribution across different frequency bands of the EEG signal, providing valuable insights into brain activity in different emotional states. The differential entropy (DE) captures the complexity of the EEG signal, serving as a measure of uncertainty associated with different data samples, which is crucial for emotion analysis.

SEED also provides features that reveal information about asymmetry between brain hemispheres. The differential asymmetry (DASM) technique provides a measure of the difference in electrical activities between the left and right hemispheres, while rational asymmetry (RASM) and asymmetry (ASM) measure imbalances in brain activities between specific pairs of electrodes. These features are of great importance as many studies have linked hemispheric asymmetries to different emotional states [13,33,34].

Additionally, the SEED dataset includes differential causality (DCAU), which explores the causal relationships between different brain regions. This feature provides insights into how different brain areas interact and influence each other, which is crucial for understanding the neural basis of emotions. Understanding the fundamental characteristics of electroencephalogram (EEG) signals, particularly their frequency and amplitude, is crucial for accurate emotion analysis as these signals reflect brain activity and exhibit variations linked to different mental states. The traditional frequency bands—delta (0.5–4 Hz), theta (4–8 Hz), alpha (8–13 Hz), beta (13–30 Hz), and gamma (30–100 Hz)—each correspond to specific neural functions, with delta associated with deep sleep, alpha with a relaxed state, beta with active mental engagement, and gamma with heightened cognitive processing. Amplitude, which indicates the intensity of neural activity, varies with mental states; for example, higher alpha amplitudes occur during relaxation, while beta amplitudes increase during intense cognitive tasks. The interplay between frequency and amplitude provides insights into distinct emotional and cognitive states, enabling effective emotion classification based on EEG signals. Understanding these characteristics allows the identification of patterns within the different frequency bands, thereby enhancing emotion detection capabilities through EEG analysis.

The attributes PSD, DE, DASM, RASM, ASM, and DCAU have the following respective dimensions: 310 (62 electrodes × 5 bands), 310 (62 electrodes × 5 bands), 135 (27 pairs of electrodes × 5 bands), 135 (27 pairs of electrodes × 5 bands), 270 (54 pairs of electrodes × 5 bands), and 115 (23 pairs of electrodes × 5 bands). A summary is presented in Table 2.

#### 3.3.2. Feature Smoothing

Utilizing the method of feature smoothing through linear dynamical systems (LDS) offers a promising approach for processing electroencephalography (EEG) signals. One of the main advantages of this approach is the ability to reduce noise in EEG signals, resulting in smoother and more representative features. This is crucial as EEG signals often suffer from various types of interferences, such as muscle and electrical artifacts, which can compromise the accuracy of analyses [32]. In the case of SEED, the authors employed the LDS method to filter out components not associated with emotional states (noise and artifacts) and consider emotional states’ temporal dynamics. By smoothing features, LDS can contribute to removing these interferences, thus improving the quality of extracted information and, consequently, the accuracy of emotional analyses [5].

Furthermore, using LDS in feature smoothing allows for incorporating information from previous states, creating a more coherent temporal relationship between data. This is particularly relevant in EEG as brain activity is inherently time-dependent. Through a sequential approach, LDS considers the temporal evolution of features, providing a more dynamic representation of EEG signals and thus preserving relevant temporal information for emotion analysis [35].

However, it is essential to note that the use of LDS for feature smoothing also faces challenges. One of the main challenges is the trade-off between smoothing and preserving important information. In some cases, excessive smoothing can lead to the loss of relevant details, affecting the precision of analyses. Additionally, applying LDS requires a good understanding of the properties of the underlying dynamical system in EEG data, which can be a complex task and may require proper adjustments [36].

In summary, feature smoothing through LDS presents significant advantages for processing EEG signals, reducing noise, improving information quality, and preserving temporal information. However, it is essential to consider the challenges associated with the excessive smoothing and modelling of dynamical systems, aiming for a balance that results in more reliable and accurate emotional analyses.

### 3.4. Experimental Design

Logistic Regression (LR) and K-Nearest Neighbors (KNN) are commonly used models for EEG-based emotion recognition due to their simplicity, interpretability, and computational efficiency [12]. These models can handle high-dimensional EEG features without complex tuning, establishing a baseline of accuracy. However, they may struggle to capture complex nonlinear patterns in the EEG data. On the other hand, Support Vector Machines (SVM) can handle high-dimensional EEG features and can also capture nonlinear relationships between EEG features and emotional states using kernel functions. SVM is a widely used benchmark model in the literature for emotion recognition tasks [7]. Deep Neural Networks (DNN) can automatically learn hierarchical representations from raw EEG signals, enabling the capture of complex nonlinear patterns. DNN can leverage large-scale EEG datasets to achieve state-of-the-art performance in emotion recognition. However, DNN requires extensive computational resources, which can be a limitation for practical applications [18]. Graph Convolutional Neural Networks (DGCNN) can incorporate the spatial relationships between EEG electrodes by modeling the EEG data as a graph and also learning dynamic representations of EEG signals, capturing the temporal evolution of brain activity patterns. Compared to DNN, DGCNN has significantly reduced training time, making it more practical for applications [16]. In summary, while LR and KNN provide a simple and efficient baseline, more advanced models like SVM, DNN, and DGCNN can capture complex nonlinear patterns in EEG data and achieve higher accuracy in emotion recognition tasks, with DGCNN offering a balance between performance and computational efficiency.

A meticulous selection process was employed regarding the parameters used for each of the models. For the K-Nearest Neighbors (KNN) classifier, the Euclidean distance is chosen as the distance metric, and the number of nearest neighbours was adjusted in the range of [3,10] to find the best hyperparameter. For Logistic Regression (LR), the parameters are calculated using maximum likelihood estimation, a standard function provided by the scikit-learn module. For the Support Vector Machine (SVM) classifier, the function in the scikit-learn module with a linear kernel is adopted, and the parameter *C* is defined through grid searches in the sets $\left[2^{\left(-10\right)},2^{\left(-9\right)},\ldots ,2^{10}\right]$ and [0.1,20], with a step of 0.5 for large and small step situations, respectively.

For the DNN model used in this work, there are three hidden layers with 128, 64, and 32 hidden units, showcasing the adaptability of the model. The output layer has three units corresponding to three emotions. The non-linear activation function used is ReLU. The optimization algorithm used was RMSProp, and the number of epochs was set to 5000, demonstrating the model’s ability to handle large datasets. For the DGCNN model, the input layer corresponds to the EEG features extracted from multiple frequency bands, highlighting its versatility in handling diverse data. All other hyperparams used in each model are described in the Table 3.

After the graph filtering operation, there is a 1 × 1 convolutional layer, which aims to learn discriminative features among the various frequency domains. Additionally, to achieve the network’s non-linear mapping capability, the ReLU activation function is adopted to ensure that the outputs of the graph filtering layer are non-negative. Finally, the outputs of the activation function are fed into a multi-layer fully connected network, and a softmax function is also used to predict the desired class label information from the input EEG features.

The research was conducted on hardware running Ubuntu 20, with an Intel Core i3 4005U processor, 8 GB DDR3 RAM, and no GPUs. The software environment utilized Python 3.7.16, scikit-learn 1.0.2, and PyTorch 1.13.1.

**Figure 1:**
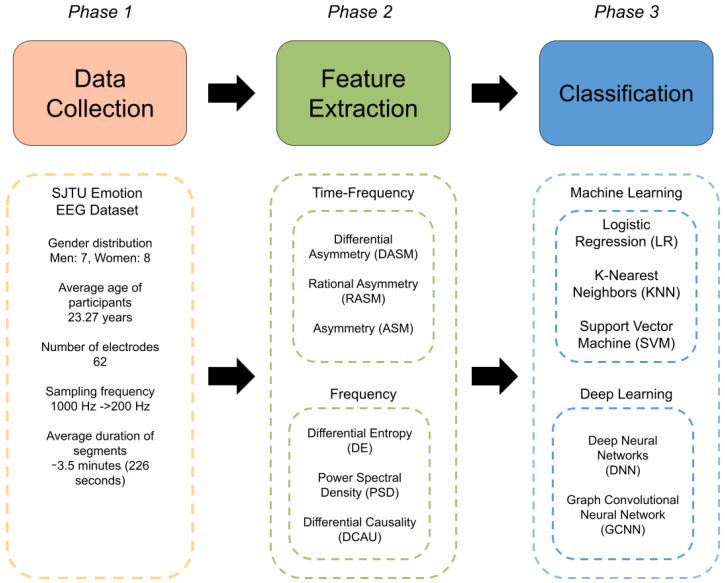
Methodology for acquisition, processing, and classification of electroencephalography data.

Figure 1 demonstrates a summary of the methodology used in this work.

#### Results Analysis

According to [6,12,13,37], there are two main types of experiments to evaluate the performance of emotion recognition in EEG signals. The first type of experiment is “subject-dependent”, while the second is “subject-independent”.

**Figure 2:**
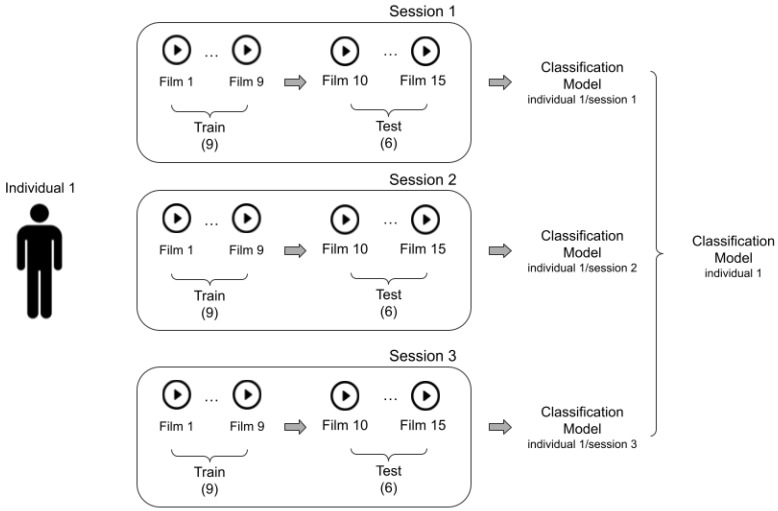
Subject-dependent approach.

In the subject-dependent approach, data from a particular subject are divided into training and testing sets. In other words, data collected from a specific subject are used to train and test the model. This allows for the evaluation of the model’s ability to recognize emotions from the subject’s EEG data. In the case of the SEED dataset, the first nine trials of EEG data are used as the training set, and the remaining six as the testing set, as shown in Figure 2. Then, the recognition accuracy corresponding to each period is obtained for each subject. Finally, the mean classification accuracy and standard deviation of all 15 subjects over two sessions are calculated. However, this approach may lead to biased results as the model may memorize specific patterns of a subject, impairing its generalization to other subjects.

On the other hand, in the subject-independent approach, data from one set of subjects are used to train the model, and data from a different set of subjects are used to test it. In this case, the model’s ability to generalize to subjects not seen during training is evaluated. This provides a more realistic assessment of the model’s performance in real-world scenarios, where it needs to recognize emotions from unknown subjects. However, this approach can be more challenging as it requires the model to capture general patterns of emotion instead of relying on specific characteristics of each subject.

In summary, the subject-dependent approach evaluates the model’s performance in recognizing emotions from a specific subject. In contrast, the subject-independent approach assesses its performance in generalizing to subjects unseen during training. Both approaches have advantages and challenges, and the choice between them depends on the study’s objectives and the nature of the EEG data. The subject-dependent approach was used in this study.

## 4. Results and Discussion

In this section, the results achieved through the application of machine learning models, namely Logistic Regression (LR), K-Nearest Neighbors (KNN), Support Vector Machines (SVM), Deep Neural Networks (DNN), and Graph Convolutional Neural Networks (DGCNN), are presented. Emotion (positive, negative, and neutral) was detected from SEED signals. Each model was evaluated considering accuracy metrics and standard deviation, providing a comprehensive view of its performance.

### 4.1. Traditional Machine Learning Models

The results presented in Table 4 demonstrate the accuracy and standard deviation for the Logistic Regression model applied to each of the five frequency bands of the EEG signal, as well as considering the entire frequency spectrum of the EEG signal. The model’s detailed results for each individual and session are shown in Table 5, Table 6, Table 7, Table 8, Table 9 and Table 10. The same results structure was generated for the *KNN* and *SVM* models.

A comparison of the performance of traditional models across different features extracted from EEG signals for emotion detection is presented in Table 11. Six features were analyzed as follows: *PSD. DE, DASM, RASM, ASM, and DCAU*, with respect to the total frequency bands. For classification, traditional machine learning models *LR*, *KNN*, and *SVM* were used.

**Figure 3:**
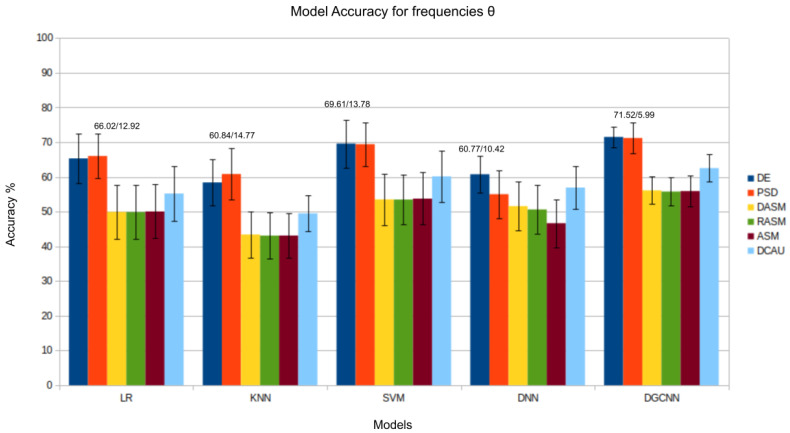
Results (accuracy/standard deviation) of all models described for frequency θ.

**Figure 4:**
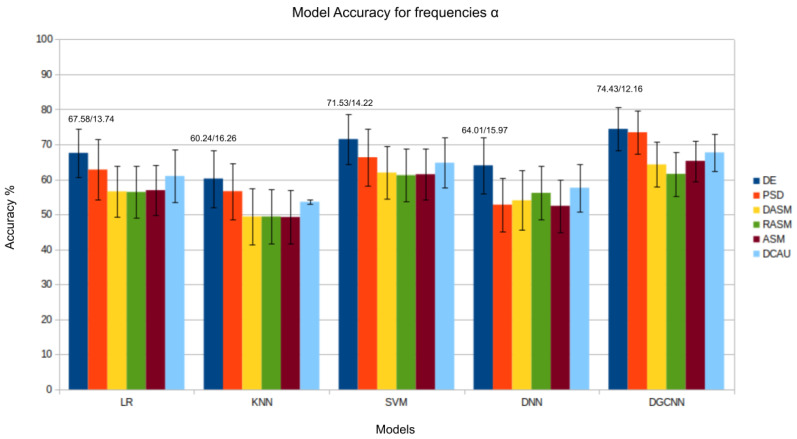
Results (accuracy/standard deviation) of all models described for frequency α.

**Figure 5:**
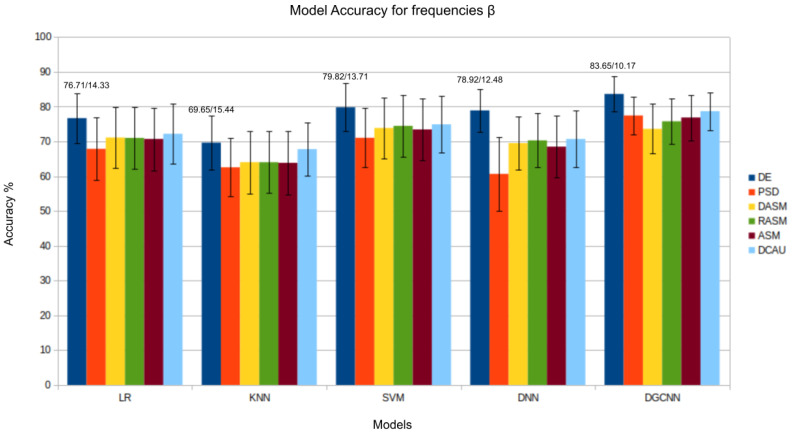
Results (accuracy/standard deviation) of all models described for frequency β.

**Figure 6:**
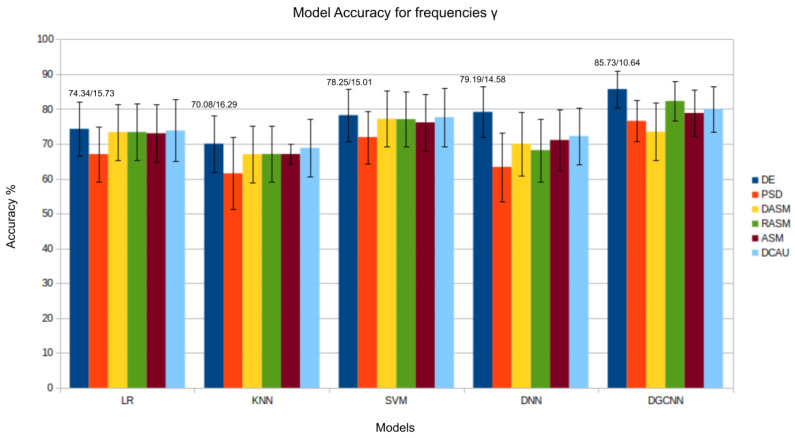
Results (accuracy/standard deviation) of all models described for frequency γ.

**Figure 7:**
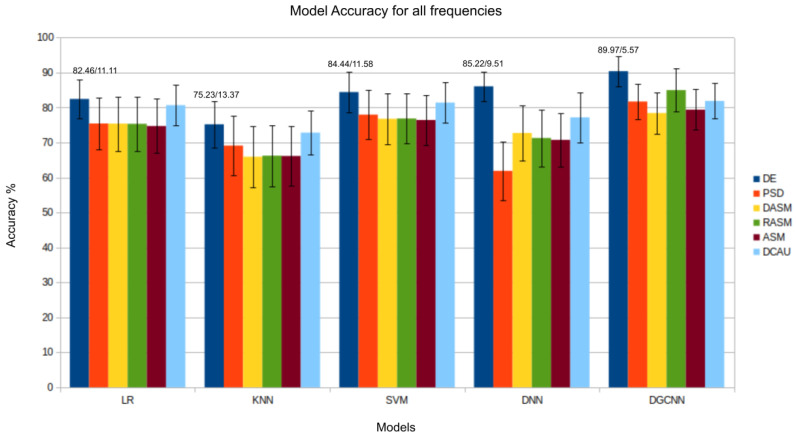
Results (accuracy/standard deviation) of all models described for all frequencies.

The results presented in Figure 3, Figure 4, Figure 5, Figure 6 and Figure 7 show that the *DE* (*differential entropy*) features exhibited higher accuracy and lower standard deviation when compared to the traditional *PSD* (*power spectral density*) features. For all three models—Logistic Regression (LR), K-Nearest Neighbors (KNN), and Support Vector Machines (SVM)—the DE features produced the highest accuracies, with respective values of 82.46%, 75.23%, and 84.44%. This result suggests that *DE* features are more suitable for emotion recognition based on EEG than other features. Another important conclusion is that the asymmetry features (*DASM*, *RASM*, *ASM*) performed similarly and, in some cases, better than PSD, despite having fewer dimensions (135 (27 pairs of electrodes × 5 bands), 135 (27 pairs of electrodes × 5 bands), 270 (54 pairs of electrodes × 5 bands), and 310 (62 electrodes × 5 bands), respectively). These results suggest that the brain processing related to positive, neutral, and negative emotions exhibits asymmetrical characteristics.

These results suggest that features such as *DE* and asymmetry features may be more relevant for emotion detection in EEG signals than traditional features like *PSD*. Additionally, the use of smoothing algorithms and the choice of the proper classifier can significantly impact the accuracy of emotion recognition.

### 4.2. Deep Learning Networks

The results in Table 12 indicate that the *DNN* classifier outperformed the *SVM* classifier, achieving an emotion recognition accuracy of 85.22%. Among the four evaluated classifiers, *DNN* proved to be the most effective for emotion recognition based on EEG. However, the processing time was a challenge due to the need to evaluate the best hyperparameters for the model. For each individual/session, 20 learning rates were evaluated, with an average processing time of 45 min for each rate, totaling 15 individuals × 3 sessions × 20 learning rates × 45 min, totaling approximately 28 h of processing. A significant advantage of the *DGCNN* model lies in its significantly reduced processing time when compared to the *DNN* model. While the *DNN* model requires an extensive training period, the *DGCNN* model achieves superior results and better accuracy in a considerably shorter time, ranging from 3 to 5 h. This efficiency in processing time enhances the practicality and feasibility of the model.

Regarding performance in each frequency band, β and γ indicate decreased activities for positive, neutral, and negative emotions. This result suggests that the spectral characteristics associated with these frequency bands are correlated with emotional expression and vary according to emotional state. This observation is consistent with the literature [12], which suggests that different emotional states may be reflected in distinct patterns of brain activity in different frequency bands.

For the *DGCNN* model, the experimental results presented in Table 12 demonstrate that the proposed method achieves better recognition performance than previous methods. The average recognition accuracy reached 89.97% for the subject-dependent experiment due to the following key points:

- The use of a non-linear neural network like *DGCNN* makes it more effective in learning non-linear discriminative features.
- The graph representation of *DGCNN* provides a useful way to characterize the intrinsic relationships between various EEG channels, which is advantageous for extracting the most discriminative features for the emotion recognition task.
- The *DGCNN* model adaptively learns the intrinsic relationships of EEG channels by optimizing the adjacency matrix *W*.

It is essential to highlight that the diagonal elements of the adjacency matrix indicate the contributions of EEG channels to emotion recognition. Therefore, the adjacency matrix can provide a way to identify which EEG channels have a more significant contribution to emotion recognition, which is advantageous for further improving emotion recognition performance.

Although the proposed *DGCNN* method has proven effective in emotion recognition from EEG, it is essential to note that the EEG databases used in the experiments are still relatively small in data volume. This may limit the use of more powerful deep neural network models and thus may restrict further improvements in the performance of this method. Therefore, it is desirable to have larger-scale EEG databases to address this challenge, which also becomes an important task for future research.

The performance of the five models is summarized in Table 13. Notably, the DGCNN model outperformed the others across all analyzed frequencies, underscoring its superiority. This result is particularly significant as it demonstrates the effectiveness of the graph-based model in five out of six analyzed attributes. However, it is worth noting that the DNN model showed higher accuracy only in the Power Spectral Density (PSD). This attribute, which captures the energy distribution across various frequencies in the EEG signal, poses a unique challenge due to its frequency distribution. Our analysis suggests that the inclusion of electrode topology mapping, achieved through the adjacency matrix in graphs, may not necessarily improve accuracy for this specific attribute.

As previously observed in this study through band analysis for traditional Machine Learning models, the β and γ bands are particularly relevant in identifying emotional states. Analyzing the β band, the *DGCNN* model’s superiority again stands out, as demonstrated in Figure 5. The *DGCNN*’s ability to handle the complexity of EEG patterns in this specific range results in a more precise interpretation of emotional responses. This consistent performance pattern highlights the model’s robustness in various emotional contexts. The Figure related to the γ band also confirms the consistency of the *DGCNN* model in presenting superior performance. Its ability to capture detailed information and nuances in EEG patterns in this frequency range underscores the crucial role of *DGCNN* in analyzing emotions in more complex neurophysiological contexts.

**Figure 8:**
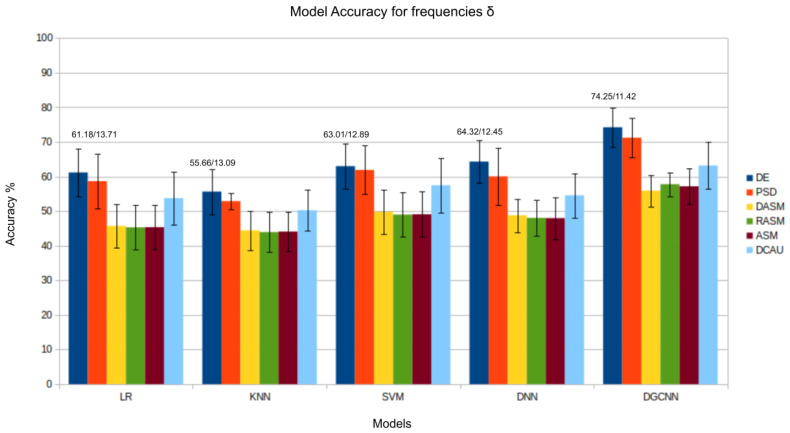
Results (accuracy/standard deviation) of all models described for frequency δ.

In the evaluation of the δ, θ, and α bands, presented in Figure 3, Figure 4 and Figure 8, respectively, it is evident that the *DGCNN* model also consistently exhibits superior accuracy, indicating its effectiveness in identifying patterns in these specific frequency ranges. However, it is crucial to note that, despite the lower accuracy compared to other bands, this decrease does not necessarily reflect a limitation of the model but rather an intrinsic characteristic of the bands themselves. The lower accuracy in these ranges suggests that δ, θ, and α may be less expressive for detecting positive, negative, and neutral emotions than other frequency bands. This underscores the importance of a contextualized interpretation of performance metrics, recognizing that different bands may play varied roles in encoding complex emotional responses. This individual analysis of the different frequency bands reinforces the reliability and overall effectiveness of the *DGCNN* model across all bands, consolidating its position as a robust choice for emotion classification in EEG signals.

Finally, a comparison with the literature is presented in Table 14, where the *SVM* and *DNN* models from the work of Zheng et al. [12] and *DGCNN* from Song et al. [38] are highlighted. These models are particularly noteworthy as they use the same dataset as this paper and, at the time of their publication, represented significant milestones in emotion classification from EEG signals. Notably, when comparing the performance of the *SVM* model across all features, the proposed model in this work exhibited higher accuracy. The improved performance is attributed to the grid search optimization conducted for each individual participant, which likely enabled the model to better capture the subject-specific patterns in the EEG data. While the literature does not specify exactly how the parameter tuning was conducted, this work verified margin adjustment through the C parameter for each individual. Another notable distinction between this work and the studies previously cited in the literature is the inclusion of the asymmetry feature (*ASM*) in conjunction with DASM and RASM. While previous works focused exclusively on *DASM* (differential asymmetry) and *RASM* (rational asymmetry) measures, the present research seeks a more comprehensive understanding of asymmetry characteristics in the EEG context. Incorporating ASM adds another dimension to the evaluation, allowing for a more refined analysis of asymmetrical characteristics in brain activity patterns. The inclusion of ASM, in conjunction with the optimization of the SVM model for each individual, highlights the innovative aspects of this work when compared to the existing literature.

The *DNN* model developed in this work stands out by significantly surpassing the *DNN* model proposed by [12] in many features, as demonstrated in Table 15. Only in the case of differential entropy was the proposed model not superior. This advancement can be attributed to specific improvements implemented in the architecture of the *DNN*, especially the learning rate for each individual of the model obtained through hyperparameter tuning, highlighting the importance of refining and adapting existing models to achieve more robust and accurate results. The surpassing of the performance of the previous *DNN* model underscores the significant contribution of this study to the advancement in the field of emotion analysis in EEG signals, promoting a deeper understanding of the capabilities and limitations of these architectures in specific contexts.

The variation in the performance metrics of the *DGCNN* model across different features, such as *DE* and *PSD* with lower accuracy and *DASM*, *RASM*, and *DCAU* with higher accuracy, when compared to the accuracy reported in the work of Song et al. [38], can be attributed to the inherent complexities of specific EEG signal characteristics. These features’ dynamic and nonlinear nature may influence the model’s behavior. For *DE* and *PSD*, which capture information about energy distribution and entropy, respectively, the DGCNN’s ability to effectively model these nuances may be related to the complexity of these representations. This is consistent with the findings of Zhang et al. [10], who observed that DE and PSD features are more challenging for deep learning models to capture when compared to asymmetry-based features. On the other hand, in features such as *DASM*, *RASM*, and *DCAU*, which reflect aspects of asymmetry and causality in the signal, the model proposed in this work was able to leverage its architecture better to identify discriminative patterns. It is important to note that despite variations in the results, the accuracy values remain close to those reported in the work of Song et al. [38], indicating consistency and validity. The convolutional layers, learning rate, and regularization parameters (weight decay) are the main factors that justify the differences between this work and the literature, and these discrepancies can be valuable for better understanding the nuances of EEG signals and providing insights into the capabilities and limitations of models in different emotional analysis contexts.

The SEED dataset, while providing a valuable resource for emotion recognition research, has two key limitations. First, the SEED dataset may not fully represent the broader population or the diverse range of emotional experiences. Its relatively small size could limit the generalizability of the models trained on it as they may not capture the full complexity and variability of human emotions. This limited representativeness could constrain the models ability to accurately classify emotions across different demographics and contexts. Second, the generalization of the results may be affected by the specific nature of the SEED dataset, which consists of Chinese audiovisual stimuli. This cultural bias could limit the applicability of the emotion classification models to diverse populations and cultural contexts [6]. Additionally, the choice of audiovisual stimuli in the SEED dataset may not capture the full range of emotional experiences, introducing a stimulus bias that could affect the models’ performance and robustness.

Furthermore, the use of a subject-dependent approach in emotion classification research with the SEED dataset, combined with its inherent limitations, introduces several potential biases that could undermine the generalizability and robustness of the models. The subject-dependent approach may lead to biased results as the model might memorize specific patterns of a subject, impairing its generalization to other subjects. This overfitting bias could result in the model performing well on the training subjects but failing to generalize to new, unseen subjects.

These potential biases, stemming from both the subject-dependent approach and the limitations of the SEED dataset, could undermine the reliability, generalizability, and real-world applicability of emotion classification research.

## 5. Conclusions

This work evaluates the effectiveness of different machine learning models, ranging from more conventional techniques to advanced deep learning approaches, in classifying emotions using EEG signals. The study seeks to identify the best-performing models and contribute to the evolution of the field, with the potential to positively impact society and technology. The novelty of this research lies in its comprehensive and comparative analysis of a wide range of machine learning and deep learning approaches, including the innovative use of Graph Convolutional Neural Networks (GCNNs), to advance emotion detection from EEG signals and address the gaps in the existing literature. It provides essential insights for emotional neuroscience, significantly expanding our understanding of the connections between brain activity and specific emotional states.

The results obtained in this study are consistent with referenced works and indicate the feasibility and potential applicability of Deep Neural Networks for emotion analysis in EEG signals. The analysis of different EEG signal features highlighted the significance of appropriate feature selection for optimal model performance. Additionally, the frequency band analysis provided a deeper understanding of EEG signal characteristics. The potential to enhance mental health systems, enabling more accurate diagnoses and personalized treatments, is noteworthy, Additionally, the possibility of using these models in brain–computer interfaces can improve human–machine interactions, making it more intuitive and efficient.

Furthermore, the results achieved through the *GCNN* architecture highlighted its effectiveness and superiority over other machine learning approaches in emotion detection from EEG. The differential entropy and differential causality attributes were the attributes that performed best in all frequency bands for the mentioned model, achieving accuracies of 89.97% and 89.36%, respectively. The *DNN* model, on the other hand, presented an accuracy of 85.22% and 84.71% for these features. Another significant result of the mentioned model concerns the *PSD* attribute, in which an accuracy of 80.56% was achieved, surpassing the *GCNN* model, with an accuracy of 79.39%. From evaluating these results, it is possible to conclude that deep learning models, especially graph-based models, are viable for emotion classification. The differential entropy and asymmetry attributes are the most suitable for correctly classifying positive, negative, and neutral emotions.

Regarding the individually analyzed frequency bands, it is notable that accuracy values are higher for the β and γ frequencies. In the γ frequency, the two highest accuracies were obtained through the *GCNN* model when analyzing the asymmetry attributes *DASM* and *RASM*, with values of 84.73% and 84.53%, confirming the relevance of the asymmetrical characteristics of EEG for emotion classification. In the β frequency, the best accuracy results were also obtained for the same model, but this time for the *DE* and *ASM* features, with values of 83.83% and 82.19%, confirming the importance of analyzing the nonlinearities of the EEG signal for emotion classification. Therefore, it is concluded that in addition to analyzing the entire frequency spectrum of the EEG signal, the analysis of specific frequency bands such as β and γ can assist in classifying emotions in EEG signals.

Given this work’s discoveries and advances, several future research directions can be explored. Investigating different network architectures, including the combination of models (Ensemble), can provide insights into optimizing the performance of DGCNN or comparing it with other deep learning models. Furthermore, exploring advanced signal processing techniques, such as adaptive filtering, can enhance the quality of features extracted from EEG signals. Analyze the data with different methodology, such as, for example, subject-independent, to explore attention mechanisms and SWAP. Investigating the generalization of models to different populations and contexts is also a critical path to ensure applicability in diverse scenarios.

## Figures and Tables

**Table 1:** Main works identifying the state of the art SOTA.

Year	Authors	Model	DATASET	Features	Highest Accuracy
2019	ZHENG, ZHU, AND LU [12]	KNN, LR, SVM, GELM	SEED	DE, PSD, DASM, RASM, DCAU	91.07%
2021	LI et al. [13]	Bi-Hemispheric Discrepancy Model -4 RNNS in 2 spatial orientations	SEED, SEED IV, MPED	DE—SEED e SEED IV STFT—MPED	SEED: 93.12% SEED IV: 74.35% MPED: 40.34%
2021	YONGQIANG [14]	LSTM + GCNN	DEAP	DE	90.45%
2022	ZHANG et al. [15]	GCB-net	SEED, DREAMER	DE, PSD, DASM, RASM, DCAU—SEED PSD—DREAMER	SEED: 92.30% DREAMER: 86.99%
2022	ZHONG, WANG, AND MIAO [4]	RGCNN	SEED, SEED IV	DE	SEED: 94.24% SEED IV: 79.37%
2023	ZHANG et al. [10]	sparse DGCNN	SEED, DEAP, DREAMER e CMEED	DE, PSD, DASM, RASM e DCAU—SEED e DEAP PSD—DREAMER, CMEED	SEED: 98.53% DEAP: 95.72% DREAMER: 92.11% CMED: 91.72%
2023	JEONG, KIN, AND KIN [16]	HSCFLM	DEAP, MAHNOB-HCI, and SEED	local and global features	DEAP: 92.10% MAHNOB-HCI: 93.3% SEED: 90.9%
2023	Zhang, X., et al. [16]	Self-training Maximum ClassifierDiscrepancy (SMCD	SEED, SEED IV	DE as 3D cube	SEED: 96.36% SEED IV: 78.49%
2023	DWIVEDI VERMA AND TARAM [18]	SPWVD	GAMEEMO	GoogleNet	84.2%
2024	FAN et al.[19]	ICaps-ResLSTM	DEAP, DREAMER	ICapsNet, ResLSTM	DEAP: 97.94% DREAMER: 94.71%
2024	ROSHDY et al. [20]	DeepFace and CNN	Local	CNN	91.21%

**Table 2:** SEED dataset features description.

Feature	Number of EEG Features per Experiment				
	δ (0.5–4 Hz)	θ (4–8 Hz)	α (8–13 Hz)	β (13–30 Hz)	γ (30–100 Hz)
PSD	62	62	62	62	62
DE	62	62	62	62	62
DASM	27	27	27	27	27
RASM	27	27	27	27	27
ASM	54	54	54	54	54
DCAU	23	23	23	23	23

**Table 3:** Hyperparameters for Machine/Deep Learning Models.

Model	Hyperparameter	Values/Description
**Logistic Regression (LR)**	Penalty	“L2”
	Solver	“lbfgs”
**K-Nearest Neighbors (KNN)**	K	3 to 10
	Distance Metric	*p* = 2 (Euclidean Distance)
**Support Vector Machine (SVM)**	Kernel	Linear
	Penalty	“L2”
	Loss	“squared hinge”
	C	Grid Search: $\left[2^{\left(-10\right)},2^{\left(-9\right)},\ldots ,2^{10}\right]$ and [0.1,20]
**Deep Neural Network (DNN)**	Hidden Layers	128 × 64 × 32
	Activation Function	ReLU
	Optimization	RMSProp
	Epochs	5000
	Learning Rate	0.007
**Graph Convolutional** **Neural Network (GCNN)**	Chebyshev Order	2
	Convolution Layer	32
	Activation Function	ReLU
	Optimization	Adam
	Epochs	20
	Learning Rate	0.01
	Weight of Decay	0.005

**Table 4:** Result (accuracy/standard deviation) of the LR model.

Model	*Feature*	δ	θ	α	β	γ	All Frequencies
LR	DE	61.18/13.71	65.32/14.16	67.58/13.74	76.71/14.33	74.34/15.73	**82.46/11.11**
	PSD	58.66/15.66	66.02/12.92	62.81/17.23	67.87/18.09	67.09/15.92	75.44/15.02
	DASM	45.31/12.83	49.85/15.55	56.42/14.7	71.02/17.92	73.41/16.25	75.34/15.56
	RASM	45.72/12.8	49.93/15.67	56.57/14.54	71.12/17.71	73.39/16.1	75.41/15.53
	ASM	45.36/12.95	50.05/15.56	56.92/14.27	70.71/17.96	73.05/16.59	74.73/15.48
	DCAU	53.77/15.15	55.22/15.72	60.98/14.92	72.19/17.31	73.86/17.74	80.69/11.72

**Table 5:** Detailed accuracy by individual/session in the LR model—*feature DE*.

Feature	Subject	Session	δ	θ	α	β	γ	All Frequencies
DE	1	1	56.58	85.19	82.15	88.22	82.15	95.3
		2	60.84	71.97	51.73	63.08	73.55	79.55
		3	45.66	69.44	53.61	61.2	56	70.09
	2	1	79.99	19.58	35.84	45.74	55.42	79.48
		2	73.99	57.73	68.64	65.03	65.61	91.84
		3	59.47	65.97	81.65	52.89	51.23	61.13
	3	1	67.92	52.96	63.37	78.83	68.28	89.88
		2	46.68	70.45	64.96	64.02	73.84	80.27
		3	63.58	74.49	90.39	91.76	87.43	97.18
	4	1	57.37	57.88	75.43	71.97	65.1	71.46
		2	59.03	56.58	59.03	58.38	63.73	68.5
		3	61.71	78.11	71.68	55.64	67.05	74.06
	5	1	67.27	88.15	61.56	55.06	37.14	70.74
		2	58.89	74.35	71.6	90.68	77.02	90.39
		3	67.12	71.97	61.78	72.83	55.71	75.14
	6	1	82.88	86.27	88.51	91.11	84.75	99.64
		2	46.32	71.6	75.29	82.73	84.1	78.03
		3	51.81	69.58	70.95	80.27	77.38	80.78
	7	1	52.1	80.71	77.67	86.85	64.31	84.03
		2	26.81	65.68	64.02	100	99.21	99.78
		3	51.95	52.17	72.04	65.82	45.88	69.87
	8	1	60.55	48.55	65.17	79.41	77.24	77.31
		2	42.27	64.02	69.58	87.21	57.44	89.02
		3	69.65	50.87	84.03	97.54	68.28	96.46
	9	1	78.83	65.46	54.91	77.96	97.4	92.77
		2	58.96	71.39	84.25	95.95	83.16	87.86
		3	55.92	53.83	62.5	61.56	71.97	67.99
	10	1	57.3	56.36	60.77	69.15	77.46	66.62
		2	69.94	34.9	41.55	66.69	68.35	70.16
		3	52.17	48.27	54.7	76.59	78.11	81.65
	11	1	48.99	65.75	79.62	64.52	76.23	70.59
		2	54.84	58.16	60.26	72.47	74.78	72.47
		3	77.82	48.63	84.68	85.69	86.2	96.03
	12	1	85.98	94.44	61.42	74.57	86.2	92.2
		2	29.91	49.78	54.48	62.07	56.07	73.27
		3	64.16	62.21	77.17	89.81	96.17	85.91
	13	1	59.75	63.87	43.06	73.92	86.05	84.39
		2	43.28	71.89	57.88	93.71	93.21	88.08
		3	69.65	86.49	55.49	87.93	83.74	80.85
	14	1	85.19	72.47	60.48	86.78	81.21	83.67
		2	64.52	70.59	62.36	72.47	68.42	79.84
		3	52.1	64.23	61.27	63.22	44.15	66.33
	15	1	70.23	72.83	91.33	93.06	98.7	100
		2	80.85	70.66	72.11	100	100	100
		3	82.44	73.05	100	97.62	100	100

**Table 6:** Detailed accuracy by individual/session in the LR model—*feature PSD*.

Feature	Subject	Session	δ	θ	α	β	γ	All Frequencies
PSD	1	1	46.24	73.7	88.8	70.09	74.71	90.17
		2	47.11	78.76	55.13	56.21	62.93	76.3
		3	35.4	52.53	58.24	71.68	60.84	54.99
	2	1	82.44	55.78	21.17	38.44	49.71	65.03
		2	72.9	55.42	60.62	49.21	60.12	81.58
		3	64.23	63.44	81.07	48.34	47.18	64.16
	3	1	61.92	58.67	70.23	61.92	62.43	78.61
		2	69.44	70.16	56.43	60.55	74.35	76.16
		3	54.26	65.82	92.34	93.93	78.83	93.42
	4	1	39.31	67.7	63.8	54.12	65.03	78.47
		2	72.9	73.05	66.69	68.21	69.73	75.51
		3	50.29	71.68	73.84	45.3	54.7	72.25
	5	1	63.29	82.01	67.63	52.75	43.79	66.11
		2	57.51	71.97	62.72	84.68	74.42	82.95
		3	69.15	63.87	49.57	70.45	59.03	73.99
	6	1	70.45	81.36	57.51	73.63	67.05	85.04
		2	40.17	48.48	44.22	45.66	45.88	37.64
		3	52.96	63.01	42.92	50.65	75.14	55.78
	7	1	51.23	76.01	71.39	60.19	44.58	72.98
		2	27.6	85.77	56.72	96.24	88.29	95.38
		3	66.26	53.83	62.36	35.4	34.47	64.09
	8	1	65.25	33.67	56.5	82.51	66.33	65.82
		2	64.96	62.21	60.77	79.77	62.43	75.94
		3	69.94	69.08	60.48	94.58	69.8	85.69
	9	1	71.75	66.55	60.04	71.53	71.1	84.32
		2	64.81	58.24	69.65	83.96	64.67	71.46
		3	59.75	48.63	79.19	61.49	61.34	69.94
	10	1	50.43	45.74	56.65	60.69	39.96	59.68
		2	45.3	58.16	39.88	57.88	52.75	43.86
		3	63.73	55.49	26.81	45.95	53.25	62.21
	11	1	28.97	65.75	83.89	54.34	82.44	86.71
		2	48.41	78.4	65.1	77.96	75.51	67.77
		3	63.44	65.61	94.08	91.26	86.2	98.77
	12	1	86.78	94.44	54.55	75.72	71.24	97.54
		2	25.79	35.04	40.68	28.47	46.6	42.2
		3	38.73	69.94	41.47	75.29	88.44	70.52
	13	1	70.81	54.12	53.83	64.02	86.27	87.21
		2	41.47	66.11	52.75	81.07	84.1	80.06
		3	59.83	78.83	57.08	84.47	67.12	77.96
	14	1	60.33	68.93	51.01	73.27	79.55	77.89
		2	54.19	83.45	67.05	79.55	62.64	74.57
		3	53.97	70.09	70.38	52.46	57.66	74.28
	15	1	86.78	71.89	91.69	91.76	97.62	100
		2	89.45	86.71	89.74	100	100	100
		3	79.84	70.66	100	98.48	98.63	100

**Table 7:** Detailed accuracy by individual/session in the LR model—*feature DASM*.

Feature	Subject	Session	δ	θ	α	β	γ	All Frequencies
DASM	1	1	59.97	49.71	68.06	86.42	96.1	96.82
		2	40.25	73.34	32.23	63.66	57.66	73.48
		3	46.82	52.46	36.13	47.47	56.43	72.04
	2	1	34.32	27.24	47.04	65.46	68.35	75.29
		2	46.82	24.86	42.85	76.16	59.54	74.78
		3	52.89	23.55	61.92	41.91	47.33	62.57
	3	1	50.72	45.74	54.34	52.82	58.82	66.76
		2	75.94	61.34	32.37	47.62	64.67	64.88
		3	56.5	51.3	56.5	78.03	75.07	96.32
	4	1	41.4	50.94	65.75	69	69.29	68.71
		2	46.24	25.72	43.57	57.37	59.61	46.6
		3	68.35	32.95	63.29	42.2	57.95	56.58
	5	1	32.59	63.87	35.69	41.62	29.7	45.01
		2	51.73	64.74	56.21	50.87	74.13	69.94
		3	49.42	32.51	35.62	40.03	52.96	57.01
	6	1	28.76	39.52	64.38	97.54	88.15	97.4
		2	17.77	55.78	59.39	65.39	72.04	61.71
		3	32.8	71.6	66.26	84.68	80.13	78.32
	7	1	45.38	70.81	72.69	74.64	78.68	84.32
		2	31.65	63.58	75.22	94.15	96.6	96.89
		3	40.46	37.36	66.26	50.79	50.94	71.03
	8	1	59.25	17.2	32.51	77.6	84.83	63.95
		2	48.41	55.64	48.34	100	72.83	91.33
		3	35.4	52.96	56.65	65.39	93.64	77.17
	9	1	62.43	66.62	53.25	75.29	68.5	74.13
		2	25.14	62.86	71.89	85.69	87.79	87.14
		3	53.32	46.68	72.98	48.55	68.64	68.06
	10	1	53.03	48.41	78.47	71.6	66.62	59.1
		2	56.21	35.19	49.93	61.71	78.68	75.94
		3	52.82	38.58	39.81	71.97	76.81	73.41
	11	1	47.9	35.91	82.37	77.53	85.12	90.61
		2	15.97	42.34	52.38	73.84	86.49	60.62
		3	58.02	32.15	84.47	97.76	89.81	93.42
	12	1	39.6	59.47	65.46	76.23	85.98	89.88
		2	40.17	27.6	50.94	48.7	39.23	40.03
		3	63.73	53.25	45.66	92.34	99.35	99.06
	13	1	38.58	74.78	50.51	79.48	91.98	92.63
		2	32.01	52.1	67.92	92.92	72.83	69.73
		3	53.97	57.73	52.1	86.85	91.47	77.38
	14	1	44	61.05	49.13	69.29	75.72	72.83
		2	59.68	77.38	50.14	77.38	75.43	74.78
		3	41.62	57.08	56.65	62.07	58.82	57.88
	15	1	29.48	44.44	40.46	88.29	86.99	91.4
		2	51.3	72.54	75.36	92.2	74.64	96.32
		3	44.36	56.14	82.44	100	96.32	100

**Table 8:** Detailed accuracy by individual/session in the LR model—*feature RASM*.

Feature	Subject	Session	δ	θ	α	β	γ	All Frequencies
RASM	1	1	65.46	52.75	67.34	85.4	95.95	97.25
		2	38.15	73.92	32.37	63.8	57.88	73.41
		3	46.24	51.66	35.84	46.1	57.51	72.69
	2	1	32.37	26.45	46.1	65.03	68.57	76.73
		2	47.62	26.66	40.97	76.01	57.95	73.05
		3	54.26	26.08	61.56	39.09	46.17	62.57
	3	1	51.37	40.17	51.73	50.07	56.43	63.22
		2	74.78	60.4	33.53	49.21	68.86	66.84
		3	58.96	49.42	55.71	78.68	73.84	96.32
	4	1	39.38	59.75	63.01	67.41	64.81	67.49
		2	44.94	24.78	51.45	56.94	62.21	50.29
		3	62.79	30.49	63.08	40.1	55.27	55.2
	5	1	36.05	62.5	34.18	40.75	26.16	44.08
		2	52.1	58.31	55.92	52.46	73.7	69.73
		3	53.03	30.85	31.79	41.62	56.79	55.56
	6	1	25.72	37.14	69.65	98.05	86.63	98.12
		2	19.15	57.08	59.32	64.31	64.88	60.77
		3	27.6	75.07	67.27	84.97	82.37	77.89
	7	1	43.5	69.51	75.29	77.24	77.67	83.89
		2	38.73	65.53	75.43	94.15	96.53	96.82
		3	38.87	35.69	64.09	51.95	50.94	72.47
	8	1	57.95	19.51	30.56	80.27	85.91	64.81
		2	50.29	52.67	48.7	99.49	77.1	91.69
		3	35.98	48.41	57.01	65.53	93.57	77.1
	9	1	61.34	67.99	53.18	73.63	70.01	74.57
		2	24.57	57.95	70.81	84.61	87.64	86.49
		3	48.05	51.01	58.02	49.28	68.14	66.04
	10	1	51.81	48.55	82.66	69.94	64.52	58.82
		2	59.03	38.95	52.53	63.22	79.12	77.1
		3	51.01	38.95	41.47	69.44	75.58	72.11
	11	1	51.59	36.71	81	77.24	86.49	90.46
		2	14.67	43.06	54.19	73.48	86.34	61.99
		3	55.06	32.08	83.67	97.62	90.39	92.56
	12	1	35.69	59.54	64.88	76.95	85.84	89.45
		2	39.31	27.67	48.63	48.84	43.64	40.03
		3	57.51	51.3	43.86	92.34	100	99.13
	13	1	42.12	72.76	48.92	79.77	92.05	91.76
		2	34.18	50.87	68.86	92.99	73.84	70.66
		3	50.36	58.45	45.09	87.07	92.49	77.89
	14	1	45.45	60.48	50.79	68.93	75.58	71.32
		2	62.07	78.03	49.71	78.25	72.18	77.17
		3	40.82	62.79	55.71	61.71	62.86	57.15
	15	1	25.58	45.81	51.16	88.37	89.31	91.18
		2	47.76	69.73	79.41	93.64	74.28	96.53
		3	45.52	55.85	82.59	100	95.59	100

**Table 9:** Detailed accuracy by individual/session in the LR model—*feature ASM*.

Feature	Subject	Session	δ	θ	α	β	γ	All Frequencies
ASM	1	1	66.18	49.35	67.12	86.99	97.04	97.4
		2	42.63	73.12	32.8	66.26	52.67	70.09
		3	44.29	53.47	35.19	53.11	56.58	77.53
	2	1	37.21	26.81	44.65	66.18	67.56	76.01
		2	44	30.13	45.16	74.57	58.45	71.82
		3	54.91	25.14	62.21	38.01	46.97	59.9
	3	1	52.02	41.84	53.25	51.66	58.96	62.57
		2	74.35	59.03	31.86	44.58	66.91	59.39
		3	58.53	50.65	57.66	77.96	77.02	91.47
	4	1	40.68	63.08	65.68	67.63	68.21	65.9
		2	46.53	27.67	45.81	57.3	62.28	51.16
		3	60.19	29.12	63.08	39.45	56.86	55.71
	5	1	33.74	64.09	37.07	38.01	27.67	42.77
		2	52.02	64.38	52.82	49.42	65.17	68.57
		3	50.72	30.27	33.82	43.14	52.67	54.62
	6	1	28.25	36.78	62.86	99.21	86.27	98.41
		2	19.08	55.49	59.61	69.08	70.16	57.51
		3	33.09	75.87	66.33	83.67	81.21	79.05
	7	1	44.65	70.23	71.97	74.78	77.67	86.13
		2	32.08	60.26	74.42	95.45	94.65	95.95
		3	36.05	33.53	68.71	50.43	50.94	74.57
	8	1	60.84	19.65	35.77	81.07	80.71	62.79
		2	50.87	57.51	50.72	98.63	83.67	91.84
		3	40.25	51.88	62.72	66.18	94.29	79.84
	9	1	62.43	67.7	53.68	76.81	69.8	74.57
		2	22.11	62.93	74.28	84.68	87.86	86.13
		3	50.65	56.72	65.68	49.86	65.32	70.52
	10	1	51.95	43.93	76.81	73.63	67.63	58.09
		2	59.25	38.01	52.6	61.2	80.85	79.19
		3	51.88	39.81	41.33	63.44	75.29	72.76
	11	1	49.28	36.78	81.58	74.06	86.49	91.62
		2	15.25	42.05	53.68	74.49	86.49	61.13
		3	57.51	32.95	83.45	98.7	90.25	93.79
	12	1	37.93	58.53	64.74	76.23	84.68	85.77
		2	39.45	26.01	52.53	44.08	30.85	41.55
		3	55.85	52.46	43.57	88.29	95.16	92.7
	13	1	40.61	67.27	48.19	79.12	91.69	90.32
		2	32.23	49.13	71.53	93.5	77.24	70.52
		3	54.05	58.74	53.47	84.47	88.44	75.43
	14	1	38.22	59.97	51.73	68.79	75.79	70.66
		2	60.98	79.34	50.65	79.48	73.41	75.87
		3	41.76	62.93	58.02	63.22	66.26	56.65
	15	1	22.4	45.38	41.76	86.92	87.28	88.22
		2	52.1	68.35	80.42	88.15	74.78	96.46
		3	41.98	53.83	80.56	100	97.04	100

**Table 10:** Detailed accuracy by individual/session in the LR model—*feature DCAU*.

Feature	Subject	Session	δ	θ	α	β	γ	All Frequencies
DCAU	1	1	29.19	60.91	81.65	74.21	86.27	88.01
		2	47.83	80.42	45.16	70.3	65.17	74.49
		3	49.64	69.44	71.68	68.42	32.08	80.35
	2	1	58.16	28.18	35.4	58.82	71.39	75.29
		2	64.16	42.05	57.95	56.72	69.08	75.79
		3	47.47	60.26	65.75	57.08	44.08	68.57
	3	1	52.31	49.35	55.13	55.92	61.85	81.43
		2	38.37	78.97	56.79	68.21	56.58	79.48
		3	71.03	32.73	48.7	86.34	71.82	93.79
	4	1	72.04	20.01	54.05	48.41	75.29	66.33
		2	65.39	40.75	49.78	53.11	72.11	72.18
		3	60.26	54.05	60.84	66.26	70.66	73.84
	5	1	58.45	57.08	41.04	35.48	39.96	56.79
		2	57.51	56.07	49.49	41.33	81.5	88.51
		3	66.55	50	24.71	52.96	26.08	50.36
	6	1	63.95	82.59	91.11	77.89	72.4	97.98
		2	41.98	57.23	57.73	85.98	89.16	82.66
		3	49.28	67.63	62.64	71.75	71.53	84.9
	7	1	42.77	56.65	56.94	46.03	60.84	61.78
		2	29.77	70.01	56.94	98.84	95.66	98.19
		3	25.43	34.68	61.49	52.31	55.13	68.57
	8	1	44.8	56.14	69.29	96.97	75.22	79.48
		2	35.77	70.45	71.53	100	88.51	93.57
		3	52.75	65.9	43.35	96.39	85.91	88.37
	9	1	75.72	45.01	40.97	73.48	92.56	90.61
		2	47.9	55.64	73.7	90.25	95.3	74.93
		3	66.26	44.36	69.58	51.08	51.01	65.53
	10	1	31.07	82.3	56.14	72.47	71.24	69.36
		2	77.89	35.33	58.09	68.06	82.01	80.64
		3	27.6	43.93	54.12	56.5	67.63	78.4
	11	1	62.79	65.17	84.9	71.75	78.18	77.53
		2	36.34	45.52	79.41	89.38	88.73	84.32
		3	71.39	34.1	92.34	93.42	86.49	97.18
	12	1	74.13	75.36	76.52	80.78	80.49	88.51
		2	35.26	45.59	51.3	59.68	65.61	71.39
		3	44.94	76.81	77.38	91.76	92.41	89.6
	13	1	64.02	82.08	36.27	79.91	90.9	81.94
		2	42.92	59.83	64.81	79.99	86.34	85.62
		3	68.71	59.32	60.98	88.08	93.57	96.46
	14	1	84.32	42.27	51.3	80.64	94.36	81.94
		2	51.66	56.36	59.03	65.53	50.29	90.03
		3	42.41	48.19	74.49	65.46	59.75	64.31
	15	1	55.56	32.3	59.47	71.53	90.61	83.74
		2	64.67	55.78	79.99	100	92.05	98.48
		3	69.44	58.31	73.99	98.92	95.95	100

**Table 11:** Results (accuracy/standard deviation) of the LR, KNN, and SVM models.

Model	*Feature*	δ	θ	α	β	γ	All Frequencies
LR	DE	61.18/13.71	65.32/14.16	67.58/13.74	76.71/14.33	74.34/15.73	**82.46/11.11**
	PSD	58.66/15.66	66.02/12.92	62.81/17.23	67.87/18.09	67.09/15.92	75.44/15.02
	DASM	45.31/12.83	49.85/15.55	56.42/14.7	71.02/17.92	73.41/16.25	75.34/15.56
	RASM	45.72/12.8	49.93/15.67	56.57/14.54	71.12/17.71	73.39/16.1	75.41/15.53
	ASM	45.36/12.95	50.05/15.56	56.92/14.27	70.71/17.96	73.05/16.59	74.73/15.48
	DCAU	53.77/15.15	55.22/15.72	60.98/14.92	72.19/17.31	73.86/17.74	80.69/11.72
KNN	DE	55.66/13.09	58.41/13.19	60.24/16.26	69.65/15.44	70.08/16.29	**75.23/13.37**
	PSD	52.9/14.64	60.84/14.77	56.63/16.06	62.57/16.92	61.56/20.8	69.14/16.86
	DASM	43.95/11.7	43.09/13.37	49.42/15.38	64.03/17.66	67.11/16.14	66.24/17.53
	RASM	44.43/11.41	43.41/13.27	49.42/15.8	64.04/17.96	67.05/16.25	65.94/17.35
	ASM	44.09/11.43	43.1/12.99	49.26/15.33	63.84/18.09	67.1/15.57	66.16/17.09
	DCAU	50.27/11.77	49.5/10.38	53.6/11.18	67.8/15.09	68.88/16.44	72.84/12.75
SVM	DE	63.01/12.89	69.61/13.78	71.53/14.22	79.82/13.71	78.25/15.01	**84.44/11.58**
	PSD	61.91/14.1	69.43/12.41	66.33/16.42	71.04/17.01	71.96/15.02	77.99/13.91
	DASM	48.99/12.89	53.49/14.54	61.21/14.96	74.48/17.64	77.11/15.83	76.88/14.46
	RASM	49.83/12.83	53.53/14.9	61.95/15.02	73.87/17.67	77.22/15.9	76.8/14.44
	ASM	49.09/13.04	53.76/15.03	61.48/14.58	73.44/17.71	76.17/16.07	76.45/14.37
	DCAU	57.49/15.75	60.16/14.62	64.77/14.3	74.9/16.34	77.67/16.85	81.43/11.62

**Table 12:** Results (accuracy/standard deviation) of the DNN and DGCNN models.

Model	*Feature*	δ	θ	α	β	γ	All Frequencies
DNN	DE	73.35/10.94	74.52/8.38	75.32/12.61	80.47/13.51	83.34/10.13	**85.22/9.51**
	PSD	66.97/12.13	75.00/9.45	71.05/12.43	76.99/13.41	78.94/12.4	80.56/12.35
	DASM	57.01/7.23	61.91/10.56	67.82/11.67	79.01/13.57	81.85/12.17	79.59/12.15
	RASM	56.68/9.05	60.81/11.02	66.89/13.58	79.2/13.62	81.73/12.16	79.08/12.25
	ASM	55.33/8.25	59.97/10.46	66.96/11.82	78.35/13	80.14/12.38	81.19/11.5
	DCAU	70.07/6.89	68.39/11.6	72.42/9.54	80.94/11.63	83.06/10.74	84.71/9.82
DGCNN	DE	74.81/8.41	69.81/11.6	71.62/13.71	83.83/9.4	83.62/10.76	**89.97/5.57**
	PSD	70.25/13.41	69.63/8.63	65.26/10.99	71.67/15.5	75.87/18.33	79.39/15.08
	DASM	60.47/9.53	63.23/8.72	63.15/11.68	81.81/11.91	84.73/8.72	85.86/7.41
	RASM	60.14/10.36	62.17/10.09	63.01/11.11	81.92/10.26	84.53/9.29	84.82/7.79
	ASM	62.69/8.18	61.00/9.29	62.20/11.82	82.19/11.74	83.68/10.48	85.46/9.43
	DCAU	71.18/10.85	67.69/10.12	67.08/8.96	81.98/9.72	82.20/9.22	89.36/8.63

**Table 13:** Results (accuracy/standard deviation) of all models described by feature.

*Feature*	Model	δ	θ	α	β	γ	All Frequencies
DE	LR	61.18/13.71	65.32/14.16	67.58/13.74	76.71/14.33	74.34/15.73	82.46/11.11
	KNN	55.66/13.09	58.41/13.19	60.24/16.26	69.65/15.44	70.08/16.29	75.23/13.37
	SVM	63.01/12.89	69.61/13.78	71.53/14.22	79.82/13.71	78.25/15.01	84.44/11.58
	DNN	73.35/10.94	74.52/8.38	75.32/12.61	80.47/13.51	83.34/10.13	85.22/9.51
	DGCNN	74.81/8.41	69.81/11.60	71.62/13.71	83.83/9.40	83.62/10.76	**89.97/5.57**
PSD	LR	58.66/15.66	66.02/12.92	62.81/17.23	67.87/18.09	67.09/15.92	75.44/15.02
	KNN	52.9/14.64	60.84/14.77	56.63/16.06	62.57/16.92	61.56/20.8	69.14/16.86
	SVM	61.91/14.1	69.43/12.41	66.33/16.42	71.04/17.01	71.96/15.02	77.99/13.91
	DNN	66.97/12.13	75.00/9.45	71.05/12.43	76.99/13.41	78.94/12.40	**80.56/12.35**
	DGCNN	70.25/13.41	69.63/8.63	65.26/10.99	71.67/15.50	75.87/18.33	79.39/15.08
DASM	LR	45.72/12.8	49.93/15.67	56.57/14.54	71.12/17.71	73.39/16.1	75.41/15.53
	KNN	44.43/11.41	43.41/13.27	49.42/15.8	64.04/17.96	67.05/16.25	65.94/17.35
	SVM	49.83/12.83	53.53/14.9	61.95/15.02	73.87/17.67	77.22/15.9	76.8/14.44
	DNN	57.01/7.23	61.91/10.56	67.82/11.67	79.01/13.57	81.85/12.17	79.59/12.15
	DGCNN	60.47/9.53	63.23/8.72	63.15/11.68	81.81/11.91	84.73/8.72	**85.86/7.41**
RASM	LR	45.31/12.83	49.85/15.55	56.42/14.7	71.02/17.92	73.41/16.25	75.34/15.56
	KNN	43.95/11.7	43.09/13.37	49.42/15.38	64.03/17.66	67.11/16.14	66.24/17.53
	SVM	48.99/12.89	53.49/14.54	61.21/14.96	74.48/17.64	77.11/15.83	76.88/14.46
	DNN	56.68/9.05	60.81/11.02	66.89/13.58	79.2/13.62	81.73/12.16	79.08/12.25
	DGCNN	60.14/10.36	62.17/10.09	63.01/11.11	81.92/10.26	84.53/9.29	**84.82/7.79**
ASM	LR	45.36/12.95	50.05/15.56	56.92/14.27	70.71/17.96	73.05/16.59	74.73/15.48
	KNN	44.09/11.43	43.1/12.99	49.26/15.33	63.84/18.09	67.1/15.57	66.16/17.09
	SVM	49.09/13.04	53.76/15.03	61.48/14.58	73.44/17.71	76.17/16.07	76.45/14.37
	DNN	55.33/8.25	59.97/10.46	66.96/11.82	78.35/13.00	80.14/12.38	81.19/11.50
	DGCNN	62.69/8.18	61.00/9.29	62.2/11.82	82.19/11.74	83.68/10.48	**85.46/9.43**
DCAU	LR	53.77/15.15	55.22/15.72	60.98/14.92	72.19/17.31	73.86/17.74	80.69/11.72
	KNN	50.27/11.77	49.5/10.38	53.6/11.18	67.8/15.09	68.88/16.44	72.84/12.75
	SVM	57.49/15.75	60.16/14.62	64.77/14.3	74.9/16.34	77.67/16.85	81.43/11.62
	DNN	70.07/6.89	68.39/11.60	72.42/9.54	80.94/11.63	83.06/10.74	84.71/9.82
	DGCNN	71.18/10.85	67.69/10.12	67.08/8.96	81.98/9.72	82.2/9.22	**89.36/8.63**

**Table 14:** Comparison of results with the literature.

Feature	Model	δ	θ	α	β	γ	All Frequencies
DE	LR	61.18/13.71	65.32/14.16	67.58/13.74	76.71/14.33	74.34/15.73	82.46/11.11
	KNN	55.66/13.09	58.41/13.19	60.24/16.26	69.65/15.44	70.08/16.29	75.23/13.37
	SVM	63.01/12.89	69.61/13.78	71.53/14.22	79.82/13.71	78.25/15.01	84.44/11.58
	**SVM** [12]	**60.50/14.14**	**60.95/10.20**	**66.64/14.41**	**80.76/11.56**	**79.56/11.38**	**83.99/9.72**
	DNN	73.35/10.94	74.52/8.38	75.32/12.61	80.47/13.51	83.34/10.13	85.22/9.51
	**DBN** [12]	**64.32/12.45**	**60.77/10.42**	**64.01/15.97**	**78.92/12.48**	**79.19/14.58**	**86.08/8.34**
	DGCNN	74.81/8.41	69.81/11.60	71.62/13.71	83.83/9.40	83.62/10.76	89.97/5.57
	**DGCNN** [38]	**74.25/11.42**	**71.52/5.99**	**74.43/12.16**	**83.65/10.17**	**85.73/10.64**	**90.40/8.49**
PSD	LR	58.66/15.66	66.02/12.92	62.81/17.23	67.87/18.09	67.09/15.92	75.44/15.02
	KNN	52.9/14.64	60.84/14.77	56.63/16.06	62.57/16.92	61.56/20.8	69.14/16.86
	SVM	61.91/14.1	69.43/12.41	66.33/16.42	71.04/17.01	71.96/15.02	77.99/13.91
	**SVM** [12]	**58.03/15.39**	**57.26/15.09**	**59.04/15.75**	**73.34/15.20**	**71.24/16.38**	**59.60/15.93**
	DNN	66.97/12.13	75.00/9.45	71.05/12.43	76.99/13.41	78.94/12.40	80.56/12.35
	**DBN** [12]	**60.05/16.66**	**55.03/13.88**	**52.79/15.38**	**60.68/21.31**	**63.42/19.66**	**61.90/16.65**
	DGCNN	70.25/13.41	69.63/8.63	65.26/10.99	71.67/15.50	75.87/18.33	79.39/15.08
	**DGCNN** [38]	**71.23/11.42**	**71.20/8.99**	**73.45/12.25**	**77.45/10.81**	**76.60/11.83**	**81.73/9.94**
DASM	LR	45.72/12.8	49.93/15.67	56.57/14.54	71.12/17.71	73.39/16.1	75.41/15.53
	KNN	44.43/11.41	43.41/13.27	49.42/15.8	64.04/17.96	67.05/16.25	65.94/17.35
	SVM	49.83/12.83	53.53/14.9	61.95/15.02	73.87/17.67	77.22/15.9	76.8/14.44
	**SVM** [12]	**48.87/10.49**	**53.02/12.76**	**59.81/14.67**	**75.03/15.72**	**73.59/16.57**	**72.81/16.57**
	DNN	57.01/7.23	61.91/10.56	67.82/11.67	79.01/13.57	81.85/12.17	79.59/12.15
	**DBN** [12]	**48.79/9.62**	**51.59/13.98**	**54.03/17.05**	**69.51/15.22**	**70.06/18.14**	**72.73/15.93**
	DGCNN	60.47/9.53	63.23/8.72	63.15/11.68	81.81/11.91	84.73/8.72	85.86/7.41
	**DGCNN** [38]	**55.93/9.14**	**56.12/7.86**	**64.27/12.72**	**73.61/14.35**	**73.50/16.6**	**78.45/11.84**
RASM	LR	45.31/12.83	49.85/15.55	56.42/14.7	71.02/17.92	73.41/16.25	75.34/15.56
	KNN	43.95/11.7	43.09/13.37	49.42/15.38	64.03/17.66	67.11/16.14	66.24/17.53
	SVM	48.99/12.89	53.49/14.54	61.21/14.96	74.48/17.64	77.11/15.83	76.88/14.46
	**SVM** [12]	**47.75/10.59**	**51.40/12.53**	**60.71/14.57**	**74.59/16.18**	**74.61/15.57**	**74.74/14.79**
	DNN	56.68/9.05	60.81/11.02	66.89/13.58	79.2/13.62	81.73/12.16	79.08/12.25
	**DBN** [12]	**48.05/10.37**	**50.62/14.02**	**56.15/15.28**	**70.31/15.62**	**68.22/18.09**	**71.30/16.16**
	DGCNN	60.14/10.36	62.17/10.09	63.01/11.11	81.92/10.26	84.53/9.29	84.82/7.79
	**DGCNN** [38]	**57.79/6.90**	**55.79/8.10**	**61.58/12.63**	**75.79/13.07**	**82.32/11.54**	**85.00/12.47**
ASM	LR	45.36/12.95	50.05/15.56	56.92/14.27	70.71/17.96	73.05/16.59	74.73/15.48
	KNN	44.09/11.43	43.1/12.99	49.26/15.33	63.84/18.09	67.1/15.57	66.16/17.09
	SVM	49.09/13.04	53.76/15.03	61.48/14.58	73.44/17.71	76.17/16.07	76.45/14.37
	**SVM** [12]	**-**	**-**	**-**	**-**	**-**	**-**
	DNN	55.33/8.25	59.97/10.46	66.96/11.82	78.35/13.00	80.14/12.38	81.19/11.50
	**DBN** [12]	**-**	**-**	**-**	**-**	**-**	**-**
	DGCNN	62.69/8.18	61.00/9.29	62.2/11.82	82.19/11.74	83.68/10.48	85.46/9.43
	**DGCNN** [38]	**-**	**-**	**-**	**-**	**-**	**-**
DCAU	LR	53.77/15.15	55.22/15.72	60.98/14.92	72.19/17.31	73.86/17.74	80.69/11.72
	KNN	50.27/11.77	49.5/10.38	53.6/11.18	67.8/15.09	68.88/16.44	72.84/12.75
	SVM	57.49/15.75	60.16/14.62	64.77/14.3	74.9/16.34	77.67/16.85	81.43/11.62
	**SVM** [12]	**55.92/14.62**	**57.16/10.77**	**61.37/15.97**	**75.17/15.58**	**76.44/15.41**	**77.38/11.98**
	DNN	70.07/6.89	68.39/11.60	72.42/9.54	80.94/11.63	83.06/10.74	84.71/9.82
	**DBN** [12]	**54.58/12.81**	**56.94/12.54**	**57.62/13.58**	**70.70/16.33**	**72.27/16.12**	**77.20/14.24**
	DGCNN	71.18/10.85	67.69/10.12	67.08/8.96	81.98/9.72	82.2/9.22	89.36/8.63
	**DGCNN** [38]	**63.18/13.48**	**62.55/7.96**	**67.71/10.74**	**78.68/10.81**	**80.05/13.03**	**81.91/10.06**

**Table 15:** Comparison of the DNN Model with the literature.

*Feature*	Model	δ	θ	α	β	γ	All Frequencies
DE	DNN	73.35/10.94	74.52/8.38	75.32/12.61	80.47/13.51	83.34/10.13	85.22/9.51
	DBN [12]	64.32/12.45	60.77/10.42	64.01/15.97	78.92/12.48	79.19/14.58	**86.08/8.34**
PSD	DNN	66.97/12.13	75.00/9.45	71.05/12.43	76.99/13.41	78.94/12.40	**80.56/12.35**
	DBN [12]	60.05/16.66	55.03/13.88	52.79/15.38	60.68/21.31	63.42/19.66	61.90/16.65
DASM	DNN	57.01/7.23	61.91/10.56	67.82/11.67	79.01/13.57	81.85/12.17	**79.59/12.15**
	DBN [12]	48.79/9.62	51.59/13.98	54.03/17.05	69.51/15.22	70.06/18.14	72.73/15.93
RASM	DNN	56.68/9.05	60.81/11.02	66.89/13.58	79.2/13.62	81.73/12.16	**79.08/12.25**
	DBN [12]	48.05/10.37	50.62/14.02	56.15/15.28	70.31/15.62	68.22/18.09	71.30/16.16
ASM	DNN	55.33/8.25	59.97/10.46	66.96/11.82	78.35/13.00	80.14/12.38	**81.19/11.50**
	DBN [12]	-	-	-	-	-	-
DCAU	DNN	70.07/6.89	68.39/11.60	72.42/9.54	80.94/11.63	83.06/10.74	**84.71/9.82**
	DBN [12]	54.58/12.81	56.94/12.54	57.62/13.58	70.70/16.33	72.27/16.12	77.20/14.24

## Data Availability

The SEED dataset is available at https://bcmi.sjtu.edu.cn/home/seed/seed.html (accessed on 24 November 2023).
